# Mature B-cell lymphoma with acute myelitis as the first presentation: a case report and literature review

**DOI:** 10.1186/s12883-023-03164-z

**Published:** 2023-03-17

**Authors:** Yang Fan, Gen-hui Zeng, Wei Xiao, Ju-ming Yu, Xiao-dong Zhang

**Affiliations:** 1 Department of Neurology, Affiliated Hospital of North Sichuan Medical College, Nanchong, Sichuan China; 2 North Sichuan Medical College, Nanchong, Sichuan China

**Keywords:** Acute Myelitis, Mature B-cell lymphoma, Secondary central nervous system lymphoma, Case report

## Abstract

**Background:**

Lymphomas are malignant tumors of the immune system that arise in lymphoid organs and can impact the central nervous system. However, lymphomas with acute myelitis as the first manifestation are exceedingly rare, and most of them are symptoms of spinal cord damage due to the lack of specificity in their clinical manifestations. The rate of early misdiagnosis is exceedingly high, and the prognosis is dire. Here, we report a case of mature B-cell lymphoma with acute myelitis as the first presentation and review the related literature.

**Case presentation:**

In this study, We report a case of a 70-year-old male patient with bilateral lower extremity weakness, bowel and bladder dysfunction, and recurrent fever. A paraureteral mass was seen beneath the right kidney on imaging, and the final pathological biopsy revealed: CD20 ( +), mature B-cell tumor, The patient refused to undergo additional tests to ascertain the type of lymphoma and subsequent therapy and asked to be discharged. In mid-November 2020, the patient died.

**Conclusions:**

This case report shows that patients with lymphoma can present with acute myelitis as the first symptom, especially if they have recurrent fever, that conventional treatment for myelitis is ineffective, and that tumors are considered after other causes of myelitis have been ruled out. Furthermore, a focused search for tumor-related evidence, as well as early identification and therapy, may help patients live longer lives.

## Background

Lymphomas with myelopathy as the first presentation are rare [1], with only 2 patients reported at the Mayo Clinic between 1996 and 2010 [2]. Spinal cord involvement is often manifested as low back pain, limb weakness, spasm, sensory disturbance, etc. Its clinical signs lack specificity and are hampered by prior hormone therapy, making early detection extremely challenging. This article reports a case of a lymphoma patient with acute myelitis as the first presentation. The patient only had symptoms of the spinal cord and repeated unexplained fever. It took 7 months from the outset to the diagnosis, and the patient passed away 9 months later. Although the disease's prognosis is currently very dismal, early detection and treatment of the cause may prolong the patient's life.

## Case presentation

**Fig. 1:**
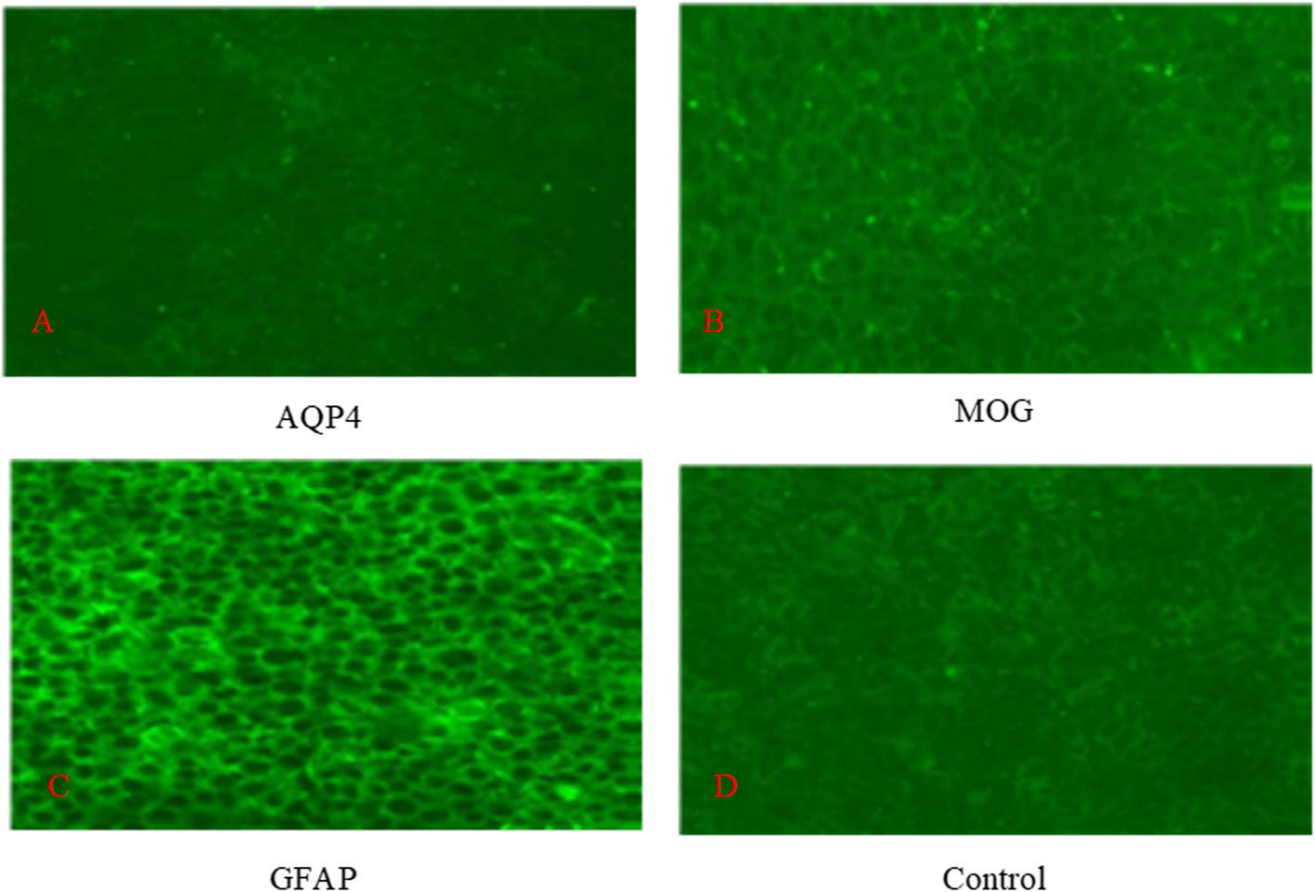
IgG results of AQP4, MOG and GFAP in serum. Serum anti-AQP4 antibody IgG levels was negative (**A**). Serum anti-MQG antibody IgG levels was negative (**B**). Serum anti-GFAP antibody IgG levels was negative (**C**). Negative control (**D**)

On August 13, 2020, a 70-year-old male was admitted to our hospital with numbness and weakness in both lower limbs, as well as dysuria.Six months prior to admission, the patient experienced progressive weakness and pain in both lower limbs with no obvious cause. The pain gradually increased, followed by standing instability and urination problems. He was taken to the community hospital for a thoracic MRI scan, which revealed a dorsal thoracic spinal cord lesion at the T2-5 vertebrae level. Examine the cerebrospinal fluid (CSF) (2020–03-16): CSF pressure was 142mmH_2_O(80-180mmH_2_O), white blood cell count was 6.0 × 10^6^/L(0.0–10.0 × 10^6^/L), total protein in the CSF was 783.0 mg/L(150.0–450.0 mg/L), glucose was 3.81 mmol/L(2.22–3.85 mmol/L), chlorine was 124.3 mmol/L(118.0–132.0 mmol/L), and bacterial smear and culture were normal. The oligoclonal band (OB) and serum anti-AQP4, MOG, and GFAP antibody IgG levels were all negative (Fig. 1). He was diagnosed with "acute myelitis" and was given a high dose of methylprednisolone (1000 mg daily for three days) followed by oral prednisone tablets (60 mg). A thoracic vertebral MRI (2020–04-01) revealed a dorsal thoracic spinal cord lesion at the level of T2-5 vertebrae, but the degree of enhancement was weaker than previously. After treatment, the patient was able to walk with crutches and was discharged from the hospital. Twenty days prior to admission, the patient experienced numbness and weakness in both lower limbs after catching a cold, which was accompanied by defecation and urination dysfunction, as well as a low fever (the highest temperature reached was 38 °C). So he went to a nearby hospital for another thoracic spinal cord MRI scan (2020–07-29), which revealed an abnormal signal in the spinal cord at the T5 vertebra level. He was given ethylprednisolone therapy (500 mg daily for 5 days) once more. The patient's temperature returned to normal after hormone withdrawal, but the rest of his symptoms remained unchanged. Past-history: He had "tuberculosis" more than 30 years ago and received formal anti-tuberculosis treatment for a year. There is no significant personal history, marriage and childbearing history, or family history. There was no history of hypertension, diabetes, or cardiopathy in the patient.

T: 36.5C, P: 80 bpm, R: 20 bpm, BP: 125/79 mmHg. Poor spirits, passive posture, no enlargement of superficial lymph nodes, and no obvious abnormalities in heart, lung, or abdominal examination. Muscle strength in both upper limbs is grade 5 and muscle tension is normal; muscle strength in the left leg is grade 3 with decreased muscle tone; and muscle strength in the right lower limb is grade 2 with decreased muscle tone. lower limbs active tendon reflex (+ + +); decreased pain sensation below the umbilical level, as well as decreased position and motor sensation in the distal right lower limb pathologic reflect( +) on both sides; There were no obvious abnormalities found during a physical examination of the residual nervous system.

CSF: Pressure was 204mmH_2_O, leukocytes were 5.0 × 10^6^/L, total protein in CSF was:1183.76 mg/L, glucose was 9.72 mmol/L, chlorine was 124.1 mmol/L, lactate was 4.30 mmol/L (0.50–2.20 mmol/L), bacterial smear and culture were normal, and OB were negative.

**Fig. 2:**
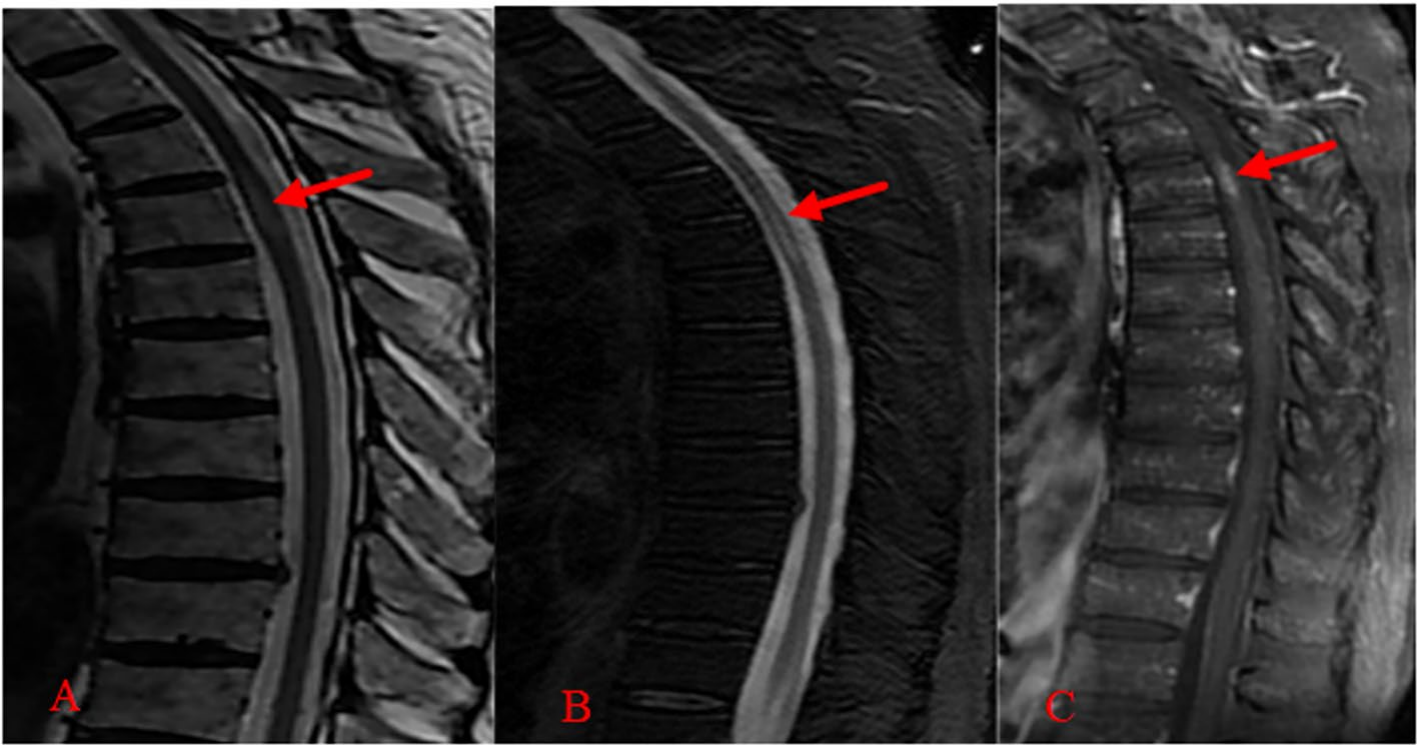
Contrast-enhanced MRI scan of thoracic vertebrae (2020–08-17). abnormal signal in the spinal cord at T5 vertebra level,Swelling of T4-6 spinal cord on T2 Flair (**A**) and T2 (**B**), with local enhancement (**C**)

**Fig. 3:**
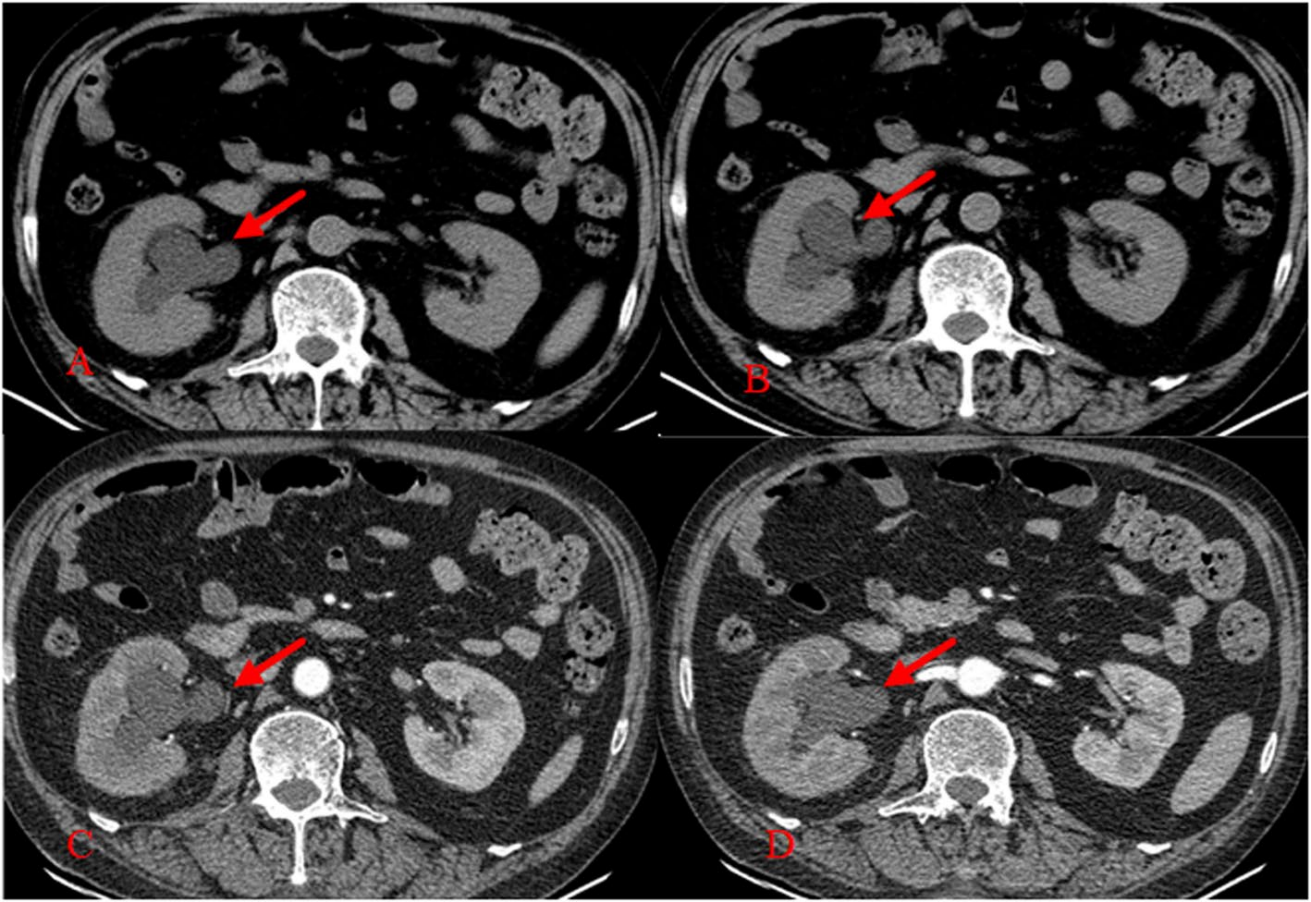
Contrast-enhanced CT scan of abdomen (2020–08-18). space-occupying lesions was located at the lower part of the ureter (about 4.8*5.7 cm in size), which was not clearly separated from the adjacent right ureter and the right psoas major muscle (**A**-**D**)

Blood: White blood cell counting:12.66 × 10^9^/L(3.50–9.50 × 10^9^/L), percentage of neutrophils:83.9%(40.0–75.0%), hemoglobin:124 g/L(130-175 g/L), hypersensitive C-reactive protein:11.13 mg/L(0-5 mg/L), albumin:27.7 g/L(40.0–55.0 g/L), potassium:3.13 mmol/L(3.50–5.30 mmol/L).Urine routine, Fecal routine, Myocardial injury markers, NT-BNP, Renal function, Blood lipids, Myeloma, Tumor markers, Connective tissue associated antibodies, Antineutrophil cytoplasmic antibody, Widal test, blood culture and coagulation function were all normal. Second liver antigen and syphilis and AIDS antibodies were negative (-). Peripheral neurologic paraneoplastic syndrome antibodies were negative. CT Scan of the Lungs: Pleomorphic changes in both lungs, considering chronic infectious lesions and the possibility of secondary pulmonary tuberculosis. The primary foci are fibrosis and calcification. Contrast-enhanced MRI scan of head, cervical vertebra and thoracic vertebrae (2020–08-17): 1. At the T4-6 level, abnormal fusiform signal was seen, with slightly low signal on T1WI and high signal on T2WI, and patchy enhancement on enhanced scan (Fig. 2) 0.2. A brain MRI scan revealed no discernible abnormalities. Contrast-enhanced CT scan of abdomen (2020–08-18): The lower ureter (lower edge of L3 to L5 vertebral level) (about 4.8*5.7 cm in size) had space-occupying lesions that were not clearly differentiated from the adjoining right ureter and right psoas major muscle. The ureter above the lesion, as well as the right renal pelvis and calyceum, were dilated and hydrocephalic, with impaired right kidney perfusion (Fig. 3).

**Fig. 4:**
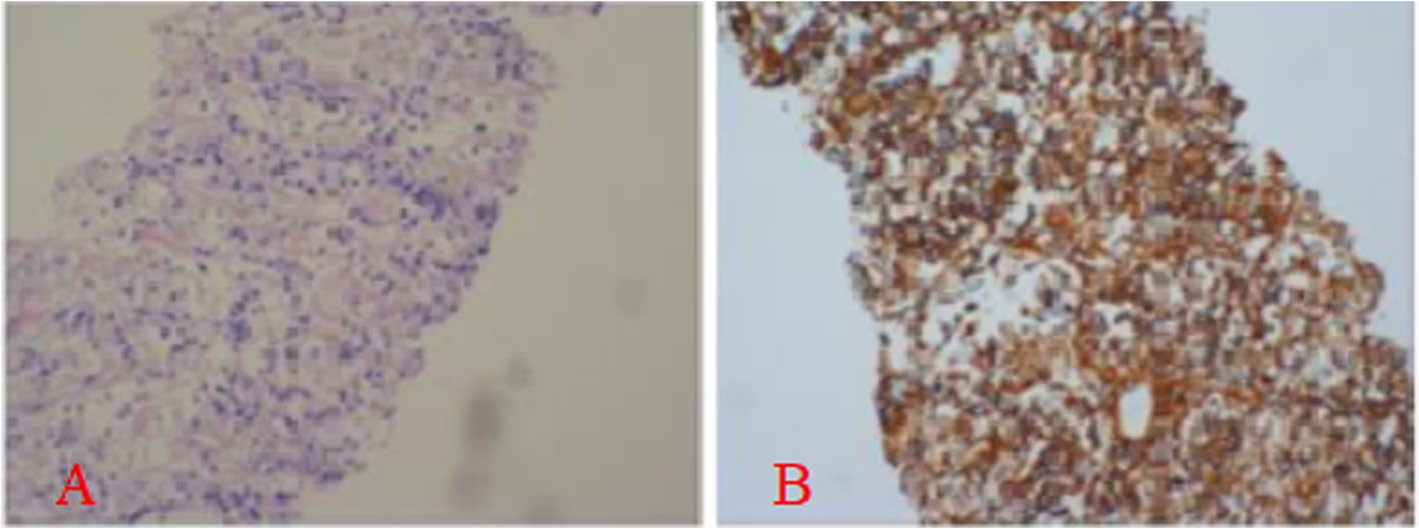
Pathological results. A: HE staining (× 200 times); B: Immunohistochemical staining, CD20( +) (× 200 times)

Localized diagnosis: T2-5 vertebral level of the dorsal thoracic spinal cord; qualitative diagnosis: myelitis. However, because the patient had previously had hormone therapy, the effect of methylprednisolone was insignificant. Meanwhile, because his blood glucose level after admission was 20.5 mmol/L, he had neurotrophic treatment as well as rehabilitative training. He developed a fever again on August 21, with a maximum temperature of 38.5 °C. For anti-infection treatment, ceftazidime (2.0 g q12h) was added. During this time, the patient experienced intermittent fever and night sweats, primarily in the afternoon and at night, with a body temperature varying between 38.5 °C and 40.3 °C. Infection related indicators were repeatedly reviewed during hospitalization: Leukocyte, neutrophil, ESR, procalcitonin and hypersensitive C-reactive protein increased. We then added Levofloxacin (0.4 g qd). On September 9, the patient received a CT-guided puncture biopsy of retroperitoneal lesions. A minor amount of coagulated necrotic material with lymphocyte infiltration is proposed. Contrast-enhanced CT scan of abdomen (2020–09-15): In comparison to CT film (2020–08-18) (4.8*5.7 cm). The lesions below the right kidney were expanded (size 5.1*6.8 cm). The final pathological findings (2020–09-18) showed that a few tiny lymphoid cells were distributed in coagulated necrotic tissue of the right nephrotic puncture tissue. CD20( +) and Ki67( +) immunohistochemical findings were obtained. With few biopsies and no additional classification, immunohistochemistry revealed a mature B-cell malignancy in the right kidney puncture tissue (Fig. 4). When combined with the immunohistochemistry data, the patient was diagnosed with a mature B-cell tumor, however the patient's family denied additional relevant examination to define the type of lymphoma and requested discharge. In mid-November 2020, the patient died.

## Discussion and conclusions

Lymphoma develops in lymph nodes and lymphatic tissues. It is an immune system malignant tumor that can spread to the central nervous system (CNS), primarily affecting the meninges and spinal cord. Lymphoma with acute myelitis as the first symptom is extremely uncommon [1]. Among 7 cases of non-Hodgkin's lymphoma (NHL) with spinal cord metastasis in The Mayo Clinic from 1996 to 2010, only 2 cases had myelopathy as the initial manifestation, and the time from the onset of spinal symptoms to the diagnosis of NHL was 1 month and 5 months, respectively [2].

Secondary central nervous system lymphoma (SCNSL) usually has a subacute onset. Low back pain, limb weakness, spasm, and sensory disturbance are all symptoms of spinal cord involvement. Pathological biopsy is the gold standard for diagnosis due to the lack of specificity of clinical symptoms. However, because to the invasive nature of pathological biopsy and the interference of prior hormone treatment, the number of verified cases depending on pathological biopsy is rather low in the actual world, making SCNSL diagnosis exceedingly difficult. In 7 Mayo Clinic patients, the median period from symptom start to final diagnosis was 6 months [2]. Another retrospective investigation found that the duration between symptom development and diagnosis ranged from 4 to 18 months [3]. The diagnosis in our instance was likewise quite tough. It took 7 months from the beginning of symptoms until the diagnosis. Initially, the patient solely displayed spinal cord problems. Meanwhile, the patient's symptoms and imaging findings improved following hormone treatment. As a result, he was diagnosed with acute myelitis. However, the patient's spinal cord symptoms worsened and he had a fever of unknown origin again after his symptoms eased. He had a TB history, and a CT scan of the lungs revealed that secondary pulmonary tuberculosis was a possibility. As a result, we first investigate if the reason of his fever is TB infection. We performed further necessary auxiliary examinations but discovered no indication of TB infection. Furthermore, anti-infection medication had a poor effect. Following that, we investigated if the patient's fever was related to space-occupying lesions below the right kidney. As a result, we did a puncture biopsy, which revealed that the tumor was mature B-cell lymphoma. Furthermore, the patient experienced only spinal cord symptoms, recurring fever, and space-occupying lesions under the right kidney throughout the course of the disease, with no weight change, splenomegaly, cytopenia, or other usual lymphoma signs, complicating the diagnosis.

The patient's myelopathy could be caused by lymphoma-induced inflammation or lymphoma metastasis, but other explanations cannot be ruled out. First of all,the patient's spinal cord lesions were smaller than before, his symptoms decreased, and the spinal inflammation and lymphoma metastases may have been improved due to the anti-inflammatory action of hormone therapy [4, 5]. The patient's spinal cord MRI, on the other hand, revealed isolated, swelling-enhanced lesions similar to the typical MRI findings of NHL secondary spinal involvement [2]. Second, the CSF and serum AQP4, MOG, and OB antibodies were negative, indicating a lower likelihood of inflammatory demyelinating lesions. Also, the patient's serum paraneoplastic syndrome 14 antibody tests came back negative, and the likelihood of paraneoplastic-associated encephalitis was low.It has been reported that the incidence of paraneoplastic disease in lymphoma is < 1%, and it is more common in Hodgkin lymphoma [6]. Of course, antibody-negative paraneoplastic encephalitis cannot be ruled out altogether.

Unfortunately, the patient's family declined further diagnosis and treatment, the nature of the myelopathy was unknown due to the lack of a pathological biopsy of the spinal cord, and the patient's lymphoma type could not be further confirmed.

According to several studies, the majority of mature B-cell lymphomas that metastasized to the CNS were diffuse large B-cell lymphomas [2, 4, 7, 8]. SCNSL has a dismal prognosis, with a median duration from diagnosis to death of 11.5 months. The 2-year survival rate following subsequent intramedullary illness is 33% [2]. However, statistics indicate that the 2-year survival rate of individuals with recurrence of CNS involvement is 0% [9]. This patient lived for 9 months from commencement until death. Additional treatment might prolong survival and reduce symptoms, but the prognosis is often very bad after affecting the CNS.

Although lymphoma with a spinal cord lesion as the first symptom is extremely rare in clinic, when the common causes, such as acute myelitis, inflammatory demyelination, and paraneoplastic myelopathy, are not clearly diagnosed, and the treatment effect is poor,clinicians must broaden their thinking and consider the possibility of lymphoma, particularly in patients with recurrent fever of unknown origin.

## Data Availability

Not applicable.

## Abbreviations

CNS: Central Nervous System
CSF: Cerebrospinal Fluid
NHL: Non-Hodgkin's lymphoma
SCNSL: Secondary central nervous system lymphoma
TB: Tuberculosis
OB: Ligoclonal band

## References

[1] Mauermann ML (2017). Neurologic Complications of Lymphoma, Leukemia, and Paraproteinemias[J]. Continuum.

[2] Flanagan EP, O'Neill BP, Habermann TM (2012). Secondary intramedullary spinal cord non-Hodgkin's lymphoma. J Neuro Oncol.

[3] Chen M, Arkadir D, Nachmias B (2021). Neurological misdiagnoses of lymphoma[J]. Neurological Sciences, Neurol Sci.

[4] Chen Y, Lin C, Zhang B (2019). Non-Hodgkin Lymphoma With Longitudinally Extensive Transverse Myelopathy as the Initial Symptom: A Case Report. Front Oncol.

[5] Elavarasi A, Dash D, Warrier AR (2018). Spinal cord involvement in primary CNS lymphoma. J Clin Neurosci.

[6] Graus F, Ariño H, Dalmau J (2014). Paraneoplastic neurological syndromes in Hodgkin and non-Hodgkin lymphomas[J]. Blood.

[7] Takahashi I, Kanoh T (2012). Case of intravascular lymphoma with a longitudinal spinal lesion diagnosed by multiple biopsies. Rinsho Shinkeigaku.

[8] Tomita, Koike, Kawagashira (2013). Clinicopathological features of neuropathy associated with lymphoma. Brain.

[9] Bernstein S H, Unger J M, Leblanc M (2009). Natural history of CNS relapse in patients with aggressive non-Hodgkin's lymphoma: a 20-year follow-up analysis of SWOG 8516 -- the Southwest Oncology Group. J Clin Oncol Official J Am Soc Clin Oncol.

